# Revitalizing myocarditis treatment through gut microbiota modulation: unveiling a promising therapeutic avenue

**DOI:** 10.3389/fcimb.2023.1191936

**Published:** 2023-05-16

**Authors:** Jingyue Wang, Xianfeng Zhang, Xinyu Yang, Hang Yu, Mengmeng Bu, Jie Fu, Zhengwei Zhang, Hui Xu, Jiachun Hu, Jinyue Lu, Haojian Zhang, Zhao Zhai, Wei Yang, Xiaodan Wu, Yan Wang, Qian Tong

**Affiliations:** ^1^ Department of Cardiovascular Medicine, The First Hospital of Jilin University, Changchun, China; ^2^ State Key Laboratory of Bioactive Substance and Function of Natural Medicines, Institute of Materia Medica, Chinese Academy of Medical Sciences/Peking Union Medical College, Beijing, China; ^3^ Department of Neurosurgery, The First Hospital of Jilin University, Changchun, China

**Keywords:** myocarditis, gut microbiota, metabolites, immune system, treatment

## Abstract

Numerous studies have demonstrated that gut microbiota plays an important role in the development and treatment of different cardiovascular diseases, including hypertension, heart failure, myocardial infarction, arrhythmia, and atherosclerosis. Furthermore, evidence from recent studies has shown that gut microbiota contributes to the development of myocarditis. Myocarditis is an inflammatory disease that often results in myocardial damage. Myocarditis is a common cause of sudden cardiac death in young adults. The incidence of myocarditis and its associated dilated cardiomyopathy has been increasing yearly. Myocarditis has gained significant attention on social media due to its association with both COVID-19 and COVID-19 vaccinations. However, the current therapeutic options for myocarditis are limited. In addition, little is known about the potential therapeutic targets of myocarditis. In this study, we review (1) the evidence on the gut-heart axis, (2) the crosslink between gut microbiota and the immune system, (3) the association between myocarditis and the immune system, (4) the impact of gut microbiota and its metabolites on myocarditis, (5) current strategies for modulating gut microbiota, (6) challenges and future directions for targeted gut microbiota in the treatment of myocarditis. The approach of targeting the gut microbiota in myocarditis is still in its infancy, and this is the study to explore the gut microbiota-immune system-myocarditis axis. Our findings are expected to pave the way for the use of gut microbiota as a potential therapeutic target in the treatment of myocarditis.

## 1 Introduction

Myocarditis is an inflammatory disease, characterized by the infiltration of inflammatory cells and deterioration of cardiac function (Richardson et al., 1996; Leuschner et al., 2015). Inflammation of the heart was first described in 1749, and the term myocarditis was coined in 1837. Furthermore, in 1980, the World Health Organization/International Society and Federation of Cardiology Task Force proposed a way of differentiating myocarditis from other myocardial diseases (Richardson et al., 1996). Myocarditis has multifaceted etiology, including infectious causes such as viral, bacterial, fungal, and parasitic infections, and non-infectious causes such as drug, autoimmune, and allergic reactions, amyloidosis, thyrotoxicosis, and genetic predisposition (Wiltshire et al., 2011; Basso, 2022). The clinical manifestations of myocarditis can vary from asymptomatic or subclinical/clinical symptoms to sudden death due to the damage of cardiomyocytes, inflammatory reaction, and myocardial fibrosis. Therefore, myocarditis poses a significant threat to the health and well-being of patients (D’Ambrosio and D’Ambrosio, 2001; Ammirati et al., 2020).

According to a previous study, cases of myocarditis have been associated with coronavirus disease 2019 (COVID-19) and COVID-19 vaccination (Inciardi et al., 2020; Heymans et al., 2022). The underlying mechanisms of myocarditis related to COVID-19 are believed to involve both direct harm caused by the virus itself and cardiac damage resulting from the host’s immune reaction (Siripanthong et al., 2020). Consequently, the management of myocarditis is an attractive area for research (Heymans et al., 2022). The primary management goals of myocarditis include alleviating biventricular load, ensuring adequate systemic and coronary perfusion, and reducing venous congestion. The management goals aim to minimize the risk of multiorgan dysfunction and accelerate recovery, transplantation, or the use of durable assist devices (Basso, 2022). Myocarditis represents a diverse group of diseases with distinct immunophenotypes. Despite extensive research and an improved understanding of the pathogenesis of myocarditis, translating knowledge into effective therapeutic strategies remains a challenge (Heymans et al., 2016). The typical management of myocarditis includes drug therapy, mechanical circulatory support, and management of complications and co-morbidities (Cooper, 2009). In addition, the management of myocarditis also includes vitamins and nutritional support. Drug therapy is the most used treatment modality for myocarditis. However, some drug therapies are ineffective and associated with severe side effects. On the other hand, mechanical circulatory support is a costly intervention and is not suitable for all patients. Moreover, immune modulation is a novel treatment approach that requires further research (Basso, 2022). Some potential strategies being explored include the use of probiotics, prebiotics, and synbiotics to modulate the gut microbiota, as well as fecal microbiota transplantation (FMT) to restore a healthy microbial balance (Schneiderhan et al., 2016; Hu et al., 2019; Wargo, 2020; Fan and Pedersen, 2021). These approaches aim to improve the overall gut health and immune response, which may have a positive impact on myocarditis treatment outcomes. It is important to note that more research is needed to establish the efficacy and safety of these strategies in the context of myocarditis.

**Figure 1:**
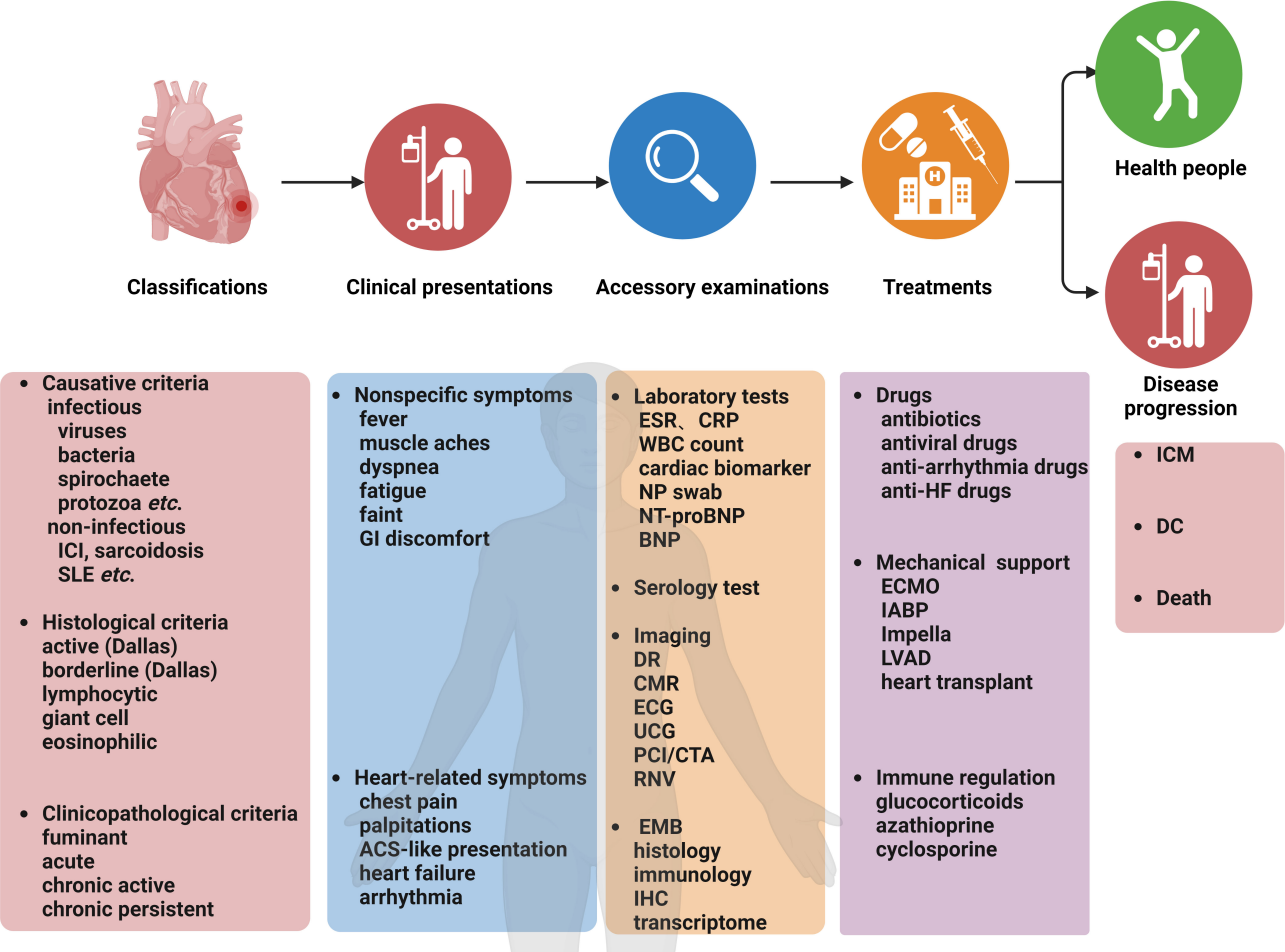
Myocarditis: Classifications, clinical presentations, accessory examinations, treatments, and prognosis. ACS, acute coronary syndrome; CMR, cardiovascular magnetic resonance; CTA, coronary computed tomography angiography; DC, dilated cardiomyopathy; DR, digital radiography; ECG, electrocardiogram; ECMO, extracorporeal membrane oxygenation; EMB, endomyocardial biopsy; FDG-PET, Fluorodeoxyglucose-positron emission tomography; GI, gastrointestinal; HF, heart failure; IABP, intra-aortic balloon pump; ICI, immune checkpoint inhibitors; IHC, immunohistochemistry; ICM, inflammatory cardiomyopathy; LVCD, left ventricular assist device; NP, nasopharyngeal; PCI, percutaneous coronary intervention; RNV, radionuclide ventriculography; SLE, systemic lupus erythematosus; UCG, ultrasound cardiography; WBC, white blood cell.

The summary of the clinical characteristics and treatment strategies for myocarditis is compiled in **Figure 1** (Trachtenberg and Hare, 2017; Błyszczuk and Błyszczuk, 2019; Leone et al., 2019; Cooper, 2021; Basso, 2022; Ammirati et al., 2023). In clinical practice, it is essential to tailor diagnostic or therapeutic approaches to individual patients, taking into account their specific circumstances and conditions. Although myocarditis has been recognized for two centuries, the available therapeutic regimens are limited (Krejci et al., 2016; Spallarossa et al., 2020). Therefore, there is a need for continued research into the pathogenesis and treatment of myocarditis to identify new therapeutic targets and cost-effective agents.

In recent years, the human gut microbiota has gained significant attention, with metagenomic studies enhancing our understanding of its diverse species and potential applications in health (Gomaa and Gomaa, 2020). The human gut microbiota contains approximately 39 trillion microorganisms and about 150 times more microbial genes (3.3x10^6) than human genes (Collins and Patterson, 2020). The large intestines have the highest microbial density, with about 100 billion bacterial cells per gram of wet stool (Sender et al., 2016). Furthermore, the gut microbiome is highly complex, with its composition varying widely between individuals. The gut microbiome comprises more than 1000 species, including bacteria, archaea, viruses, and fungi (Sekirov et al., 2010). Firmicutes, Bacteroidetes, Actinobacteria, and Proteobacteria form the most dominant bacterial phyla of the gut microbiome (Yan et al., 2022). Gut bacteria are mainly categorized into symbiotic, opportunistic, and pathogenic bacteria. Symbiotic bacteria interact with each other and with the host in a symbiotic manner. They maintain gut homeostasis and contribute to overall health (Cummings, 1983). The gut microbiome participates in various functions, including nutrient absorption, immune system regulation, and biological antagonism (Adak and Khan, 2019). However, dysbiosis of the gut microbiome has been associated with various diseases (Hou et al., 2022). The composition of the gut microbiome is affected by various factors, including genetics, diet, medication, and environment. Diet is among the most significant factors influencing the gut microbiome (Duda-Chodak et al., 2015; Singh et al., 2017; Klement and Pazienza, 2019). A high-fiber diet and plant-based foods can promote the growth of beneficial bacteria in the gut microbiome, while high-fat and high-sugar diets have been linked to dysbiosis (Koh et al., 2016; Nagai et al., 2016; Hills et al., 2019).

Previous studies have shown that the gut microbiota contributes to the occurrence and development of various cardiovascular diseases (CVDs) (Witkowski et al., 2020), including hypertension (Yang et al., 2018; Verhaar et al., 2020), atherosclerosis (Zhu et al., 2020), heart failure (HF) (Zhang et al., 2021b), arrhythmias (Oniszczuk et al., 2021), and diabetic cardiomyopathy (Bastin and Andreelli, 2020; Yuan et al., 2022). Moreover, recent studies revealed that gut microbiota was also associated with myocarditis (Gil-Cruz et al., 2019; Hu et al., 2019; Mandelbaum et al., 2020; Piccioni et al., 2021; Luo et al., 2022; Ozkan, 2022). Furthermore, gut microbiota modulation through dietary interventions, probiotics, drugs, or fecal microbiota transplantation, has shown promising results in various conditions, including inflammatory bowel disease (Li et al., 2021b), colorectal cancer (Feng et al., 2015; Wong and Yu, 2019; de Souza et al., 2022), liver diseases (Bajaj, 2019; Albillos et al., 2020), obesity (Duca et al., 2013; Gomes et al., 2018), diabetes (Qin et al., 2012; Komaroff, 2017; Hung et al., 2021), arthritis (Scher et al., 2013; Xu et al., 2022), osteoporosis (Lahiri et al., 2019; Di et al., 2021), CVDs (Ko et al., 2019; Bai et al., 2021), and neurological disorders (Varesi et al., 2022).

In recent years, the role of the gut microbiome in health and diseases has attracted significant research attention. However, animal studies and clinical trials evaluating the relationship between myocarditis and gut microbiota are limited (Hu et al., 2019). Therefore, this study reviews (1) the evidence on gut-heart axis, (2) the association between gut microbiota and the immune system, (3) the association between myocarditis and the immune system, (4) the association between gut microbiota and myocarditis, (5) current strategies for modulating gut microbiota, (6) challenges and future directions for targeted gut microbiota in the treatment of myocarditis. This study provides novel ideas for myocarditis treatment and references for future research.

## 2 Gut-heart axis: effect of gut microbiota on cardiovascular diseases

**Figure 2:**
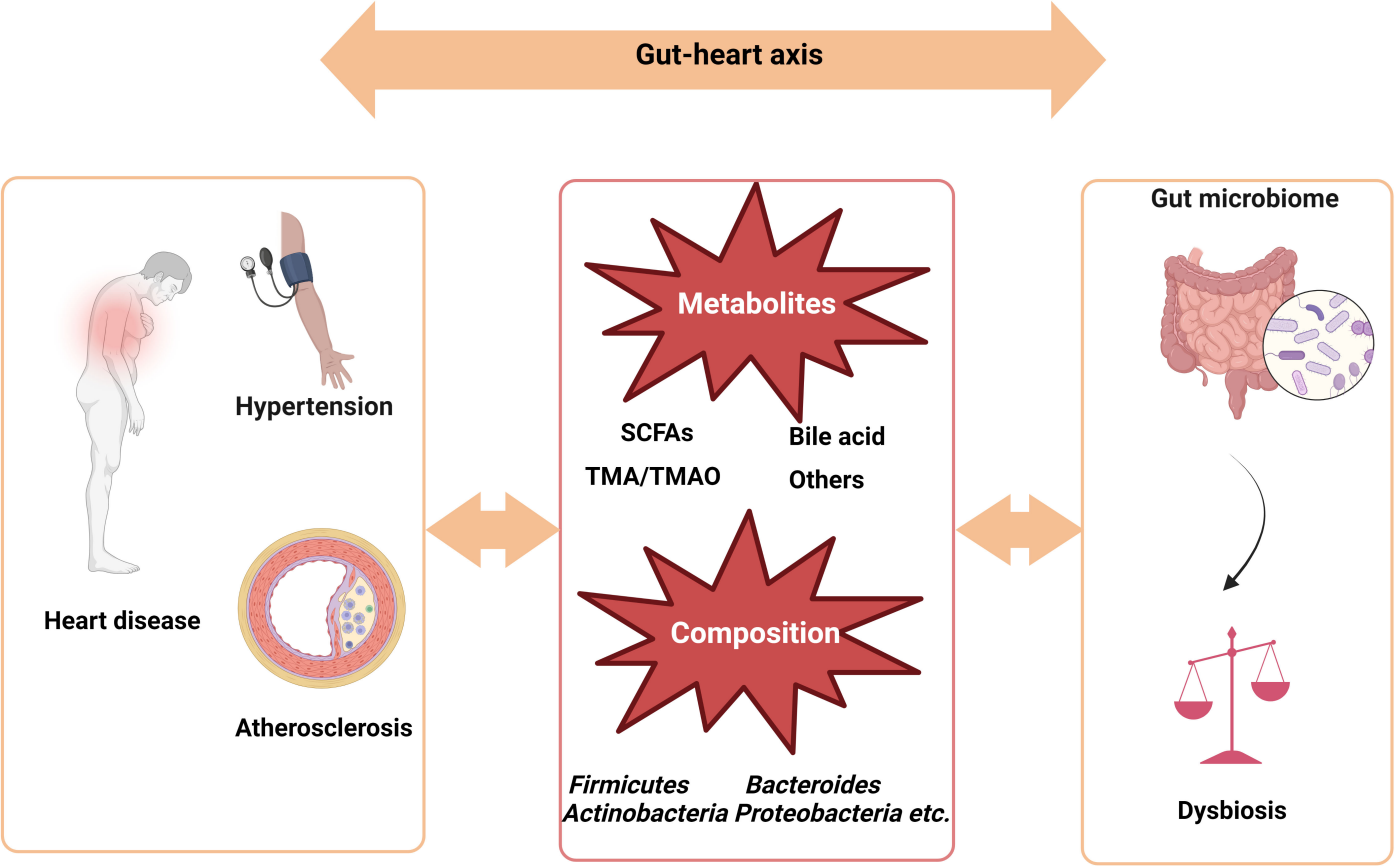
Gut-Heart axis: The relationship between gut microbiota and cardiovascular diseases. SCFAs, short-chain fatty acids; TMA, trimethylamine; TMAO, TMA-N-oxide.

The gut microbiome regulates human health. Recent studies indicate that dysbiosis of the gut microbiota is thought to be cause of most CVDs, including coronary heart disease, hypertension, and heart failure (Lippi et al., 2017; Jia et al., 2019). Gut microbiota dysbiosis can also induce an inflammatory response and affect the metabolism of bile acids (BAs), short-chain fatty acids (SCFAs), trimethylamine-N-oxide (TMAO) and other bioactive molecules, resulting in systemic inflammation and endothelial dysfunction. These changes, in turn, promote the development of atherosclerotic plaques and increase the risk of thrombosis and cardiovascular events (Liu et al., 2020b). The role of the gut-heart axis in cardiovascular health and the relationship between the gut microbiome and CVDs are presented in the subsequent sections and **Figure 2**.

### 2.1 Coronary heart disease and atherosclerosis

Coronary heart disease (CHD) is a condition in which the arteries cannot supply adequate oxygenated blood to the heart. Most cases of CHD are caused by the blockage of coronary arteries due to either atherosclerosis, thrombosis, or a combination of both (Ulbricht and Southgate, 1991).

The effects of gut microbiota in CHD are due to alterations in their composition and metabolites. One previous study enrolling 29 CHD inpatients and 35 healthy volunteers showed the proportion of phylum *Bacteroidetes* (56.12%) was lower, whereas that of the phylum *Firmicutes* was higher (37.06%) in the CHD patients than that of the healthy controls (60.92% and 32.06%, *P* <0.05) (Cui et al., 2017). Elsewhere, a similar observation was obtained (Emoto et al., 2017). Furthermore, a case-control study revealed that lower *Lactobacillus* levels are associated with an increased likelihood of severe coronary atherosclerotic lesions and myocardial necrosis as well as a poorer prognosis for patients with the acute coronary syndrome (ACS), particularly those with ST-segment elevation myocardial infarction (Gao et al., 2021). A previous study showed that *Faecalibacterium* was the dominant microorganism in the healthy control group, whereas *Escherichia-Shigella* and *Enterococcus* were enriched in the coronary artery disease group (Zhu et al., 2018). Furthermore, Yang et al. showed that hydroxyurea effectively treated atherosclerosis, reduced serum cholesterol levels, modified the gut microbiota at various levels, and affected cholesterol absorption by decreasing Niemann-Pick C1-like 1 in the epithelial cells of small intestines of apolipoprotein E knockout ApoE^(-/-)^ mice fed on a high-fat diet (Yang et al., 2022b). In addition, the severity of myocardial infarction in rats is associated with intestinal microbial metabolites (Lam et al., 2016). The gut microbiome can also affect lipid metabolism. Certain bacteria in the gut microbiome can metabolize BAs, thereby affecting lipid metabolism. On the other hand, dysbiosis can alter the production of BAs, causing changes in lipid metabolism and increasing the risk of atherosclerosis (Jonsson and Bäckhed, 2017). The gut microbiome can affect the development and progression of atherosclerosis and CHD in various ways. Firstly, inflammatory responses can exacerbate plaque development or cause plaque rupture. Secondly, cholesterol and lipids metabolism by the gut microbiota can influence the development of atherosclerotic plaques. Thirdly, diet and gut microbial metabolites, including TMAO and SCFAs, can have various effects on atherosclerosis (Jonsson and Bäckhed, 2017; Liu et al., 2020a).

### 2.2 Hypertension

Hypertension is one of the most prevalent risk factors for CVDs across the globe. Furthermore, hypertension arises due to a complex interplay of genetic and environmental factors (Li et al., 2021a; Zhou et al., 2021). Globally, hypertension and pre-hypertension account for 8.5 million deaths annually due to stroke, ischemic heart disease, other vascular disorders, and kidney diseases. The prevalence of hypertension among individuals aged 30-79 years doubled from 648 million people to 1278 million people between 1990 and 2019 (Zhou et al., 2021).

Unlike healthy controls, patients with hypertension show microbial diversity and a shift in microbial composition. In addition, the number of species associated with hypertension shows a stronger correlation with disease severity. Li et al. found that pre-hypertensive and hypertensive patients had significantly reduced microbial richness and diversity. They also exhibited distinct metagenomic composition characterized by an overgrowth of disease-associated bacteria and a decrease in healthy-associated bacteria. Additionally, these patients exhibited a *Prevotella*-dominated gut enterotype and disease-linked microbial function, in contrast to the healthy control group (Li et al., 2017). Surprisingly, the microbiome characteristics of the pre-hypertensive group were similar to those observed in the hypertensive group. In addition, a previous study enrolling 60 patients with primary hypertension and 60 gender-, age-, and body-weight-matched healthy controls revealed that opportunistic pathogenic bacteria such as *Klebsiella* spp.*, Streptococcus* spp., and *Parabacteroides merdae* were frequently found in the gut microbiome of hypertensive individuals. In contrast, beneficial bacteria producing SCFAs, such as *Roseburia* spp. and *Faecalibacterium prausnitzii*, were more abundant in the control group. Short-chain fatty acids-producing bacteria can modulate blood pressure by promoting vasodilation. In terms of microbial function, the gut microbiome of hypertensive individuals exhibited higher membrane transport, lipopolysaccharide (LPS) biosynthesis, and steroid degradation. However, the healthy controls showed higher metabolism of amino acids, cofactors, and vitamins (Yan et al., 2017). Furthermore, dysbiosis of the gut microbiome in rat models of hypertension can directly influence systolic blood pressure. Therefore, modulation of the gut microbiota can be exploited as a novel therapeutic approach for managing hypertension (Durgan et al., 2016; Adnan et al., 2017). Australian researchers recently published a review article, summarizing the latest research findings on the role of gut microbiota and their metabolic byproducts in host blood pressure regulation mechanisms. Gut microbiota imbalances can affect blood pressure regulation *via* host gene pathways, vascular function, and the autonomic nervous system. Enteric bacterial metabolites, including SCFAs and indole-3-lactic acid, are beneficial, whereas TMAO is harmful to blood pressure. Regulating gut microbiota through diet or fecal microbiota transplantation (FMT) may serve as a potential therapeutic strategy for blood pressure reduction. Moreover, the article discussed the prospects, challenges, and difficulties of using gut bacteria for blood pressure control in clinical applications (O’Donnell et al., 2023).

### 2.3 Heart failure

Heart failure is a complex clinical syndrome characterized by dyspnea and fatigue due to its inability to efficiently fill or eject blood from the heart. Heart failure can progress to pulmonary or splanchnic congestion and peripheral edema. It can be categorized into four types, including heart failure with preserved ejection fraction (HFpEF), heart failure with mildly reduced ejection fraction (HFmrEF), heart failure with reduced ejection fraction (HFrEF), and heart failure with improved ejection fraction (HFimpEF). HF is a leading cause of morbidity and mortality across the globe. The 2022 guideline offers patient-centric recommendations for healthcare professionals to prevent, diagnose, and manage patients with heart failure (Heidenreich et al., 2022).

Alteration of gut microbiota composition and microbial metabolite shifts, especially those derived from dietary nutrients increase the risk of HF. Tang et al. reported that impaired intestinal barrier function and bowel wall edema in HF patients might promote local and systemic inflammation, as well as bacterial translocation (Tang et al., 2019b). In addition, the inflammation and immune response linked to intestinal barrier impairment and bacterial translocation can exacerbate heart failure (Jia et al., 2019). Moreover, Zhang et al. showed that TMAO was involved in the pathological processes of HF and could act as a marker for identifying patients at risk of disease progression. In addition, they reviewed the gut–TMAO–HF axis as a new target for HF treatment (Zhang et al., 2021b). Romano et al. investigated the relationship between the gut microbiota-derived metabolite phenylacetylgutamine (PAGln) and HF. Clinical and mechanistic analyses reveal a dose-dependent association between circulating PAGln levels and HF presence and severity. PAGln directly promotes HF-relevant phenotypes, including decreased cardiomyocyte sarcomere contraction and elevated B-type natriuretic peptide gene expression in both cultured cardiomyoblasts and murine atrial tissue. The findings suggest that regulating the gut microbiome, particularly PAGln production, may represent a potential therapeutic target for modulating HF (Romano et al., 2023). This together suggests that innovative therapeutic approaches that target gut microbial metabolic pathways or metabolites and modify the gut microbiota composition could be effective in reducing CVDs susceptibility and preventing the progression of HF.

### 2.4 Other CVDs

In recent years, numerous studies have investigated gut microbiota as a therapeutic target for CVDs prevention and management. For example, a previous study demonstrated that *Bacteroides fragilis* could prevent aging-related atrial fibrillation (AF) in rats through regulatory T cells (Tregs) mediated regulation of inflammation. Specifically, *Bacteroides fragilis* promotes the proliferation and function of Tregs as well as reduces inflammatory responses, thus decreasing the incidence of AF (Zhang et al., 2022). These findings provide novel insights for developing new drugs to prevent or treat AF. Furthermore, a clinical study showed that SCFAs could alleviate the development of AF through G protein-coupled receptor 43/NOD-like receptor family pyrin domain containing 3 (GPR43/NLRP3) signal pathways (Zuo et al., 2022). Moreover, the potential of probiotics, prebiotics, and FMT in modulating gut microbial composition and promoting cardiovascular health has been extensively studied (Oniszczuk et al., 2021). However, further studies are needed to reveal microbial mechanisms with diagnostic and therapeutic implications in CVDs (Walker et al., 2021). Furthermore, a previous study showed that hydroxyurea could prevent diabetic cardiomyopathy by inhibiting inflammation and cell apoptosis (Zhou and Lu, 2022). Inflammation is implicated in CVDs. In addition, dysbiosis has been linked to increased inflammation. A few gut microbiota, particularly Gram-negative bacteria, produce LPS, which activate the immune system and promote inflammatory responses (Yoo et al., 2020).

In summary, the gut-heart axis represents a complex network of interactions involving the gut microbiota, their metabolites, and the cardiovascular system. Myocarditis, an important cardiovascular disease, has gained significant attention in recent years. The emergence of the gut-heart axis concept might imply a potential connection between the gut microbiota and myocarditis. By delving into the role of the gut microbiota in myocarditis, we may discover new opportunities for prevention, diagnosis, and treatment, ultimately enhancing the prognosis and quality of life for those affected.

## 3 Gut microbiota and the immune system

The immune system is divided into innate and acquired immunity. Innate immunity refers to the immune response present at birth that can produce an effective response to pathogens without previous antigen exposure (Janeway et al., 2001). Components of the innate immune system include: the skin and mucosal barriers, macrophages, natural killer (NK) cells, and the complement system. On the other hand, acquired/adaptive immunity is developed after previous exposure to pathogens or vaccinations. The components of the adaptive immune system include B lymphocytes and T lymphocytes (Janeway et al., 2001). B cells secrete antibodies that can specifically bind to and neutralize pathogens. Furthermore, T cells have different subtypes, including helper T cells and cytotoxic T cells. T cells recognize and attack the surface antigens of infected cells and coordinate the immune response (Parkin et al., 2001). The immune system can help the body to fight infections and can induce inflammation. Cytokines are a class of small proteins with broad biological activity that are synthesized and secreted by immune cells upon stimulation. They maintain the stability of the body’s immune system and regulate the occurrence of pathological processes. Cytokines are classified into pro-inflammatory and anti-inflammatory cytokines based on their effects on inflammation. Anti-inflammatory cytokines include Th2 type cytokines (IL-4, IL-5, IL-10). Pro-inflammatory cytokines include Th1 type cytokines (IFN-γ, IL-2, IL-12p70); IL-1β; IL-6, IL-8, TNF-α; and Th17 type (IL-17) (Fajgenbaum and June, 2020). Understanding the gut microflora, associations among the microbiome and inflammasomes, the immune system, the role of gut microbiota metabolites, and gut permeability may lead to the development of preventive strategies for CVDs (Noor et al., 2021).

### 3.1 Gut-immune system crosslink

Gut microbiota is involved in the regulation of host immunity. For example, *Lactobacilli* and *Bifidobacteria* improve the host immune function (Vlasova et al., 2016). Furthermore, the gut microbiota stimulate the training and development of the host immune system and the occurrence of cellular immunity, enabling the host’s innate immune system to distinguish between pathogenic and symbiotic bacteria (Wu and Wu, 2012; Ursell et al., 2014; Yoo et al., 2020). In addition, the gut microbiota colonizes, and proliferates in the intestinal mucosa, forming a layer that protects the host from invasion by foreign pathogens. In addition, gut microbiota can compete with harmful bacteria for nutrients, thereby inhibiting their growth and generating antibacterial substances that suppress the proliferation of pathogens (Belkaid et al., 2014).

**Figure 3:**
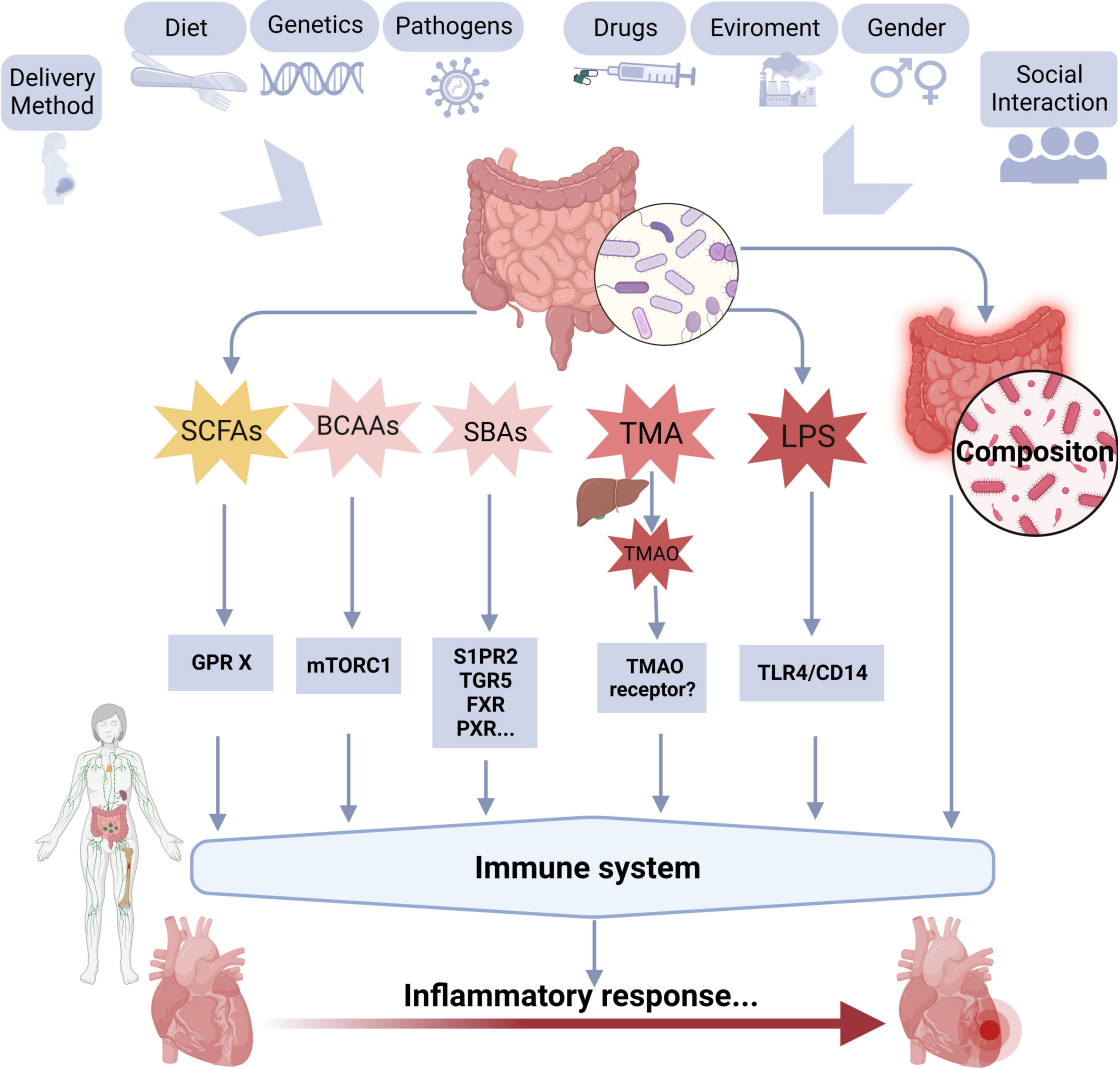
A schematic overview of the possible relationships between gut microbiota, and its metabolites, immune system, and myocarditis. BCAAs, branched-chain amino acids; GPR, G protein-coupled receptor; LPS, lipopolysaccharide; SBAs, secondary bile acids; SCFAs, short-chain fatty acids; TMAO, trimethylamine-N-oxide; TLR4, toll-like receptor 4; FXR, farnesoid X receptor, GPBAR1/TGR5, G protein-coupled bile acid receptor 1; PXR, pregnane X receptor; S1PR2, sphingosine 1-phosphate receptor 2.

Gut microbiota primarily affects the disease process through endogenous metabolites produced by gut microbiota and changes in the composition of gut microbiota. About 70~80% of the human immune cells are found in the gut, and dysbiosis of the gut microbiota is related to alterations in the immune system. Emerging evidence has focused on the role of the gut microbiota in regulating the immune response to viral infections and has shown that the gut microbiota can influence the activity of immune cells, including T cells and dendritic cells (Mizutani et al., 2022). Specifically, some gut microbiota produces metabolites that modulate the activity of immune cells and the production of pro-inflammatory cytokines, thus promoting the development of myocardial inflammation. **Figure 3** shows the possible relationship between gut microbiota and their metabolites, immune system, and myocarditis.

### 3.2 Gut microbiota composition

Gut bacteria can be classified into six primary phyla based on their genetic and physiological characteristics, including Firmicutes, Bacteroides, Actinobacteria, Proteobacteria, Fusobacteria, Verrucomicrobia, and some other phyla. Firmicutes, Bacteroides, Actinobacteria, and Proteobacteria, account for over 90% of the gut microbiota (Yan et al., 2022). (1) The phylum Firmicutes includes several common gut bacteria, such as *Lactobacillus*, *Streptococcus*, and *Clostridium*. Some *Firmicutes* species improve gut barrier function and enhance immunity (Rinninella et al., 2019). (2) The phylum Bacteroidetes include several bacteria that produce SCFAs, such as *Bacteroides fragilis*. Short-chain fatty acids have anti-inflammatory effects on the gut and promote immune homeostasis (Fabersani et al., 2021). (3) The phylum Proteobacteria includes several pathogenic bacteria, including *Escherichia coli* and *Salmonella*, which can cause gastrointestinal infections. However, some Proteobacteria, such as *Akkermansia muciniphila*, have beneficial effects on gut health, including promoting the growth of beneficial bacteria and reducing inflammation (Larsen et al., 2015). (4) The phylum Actinobacteria includes numerous beneficial gut bacteria, such as *Bifidobacterium* and *Collinsella*, which modulate the immune system, improve gut barrier function, and reduce inflammation (Barka et al., 2016).

### 3.3 Gut microbiota metabolites

Gut microbiota-dependent metabolites act as a bridge that connects the dynamic equilibrium between the host and the gut microbiota (Schoeler et al., 2019). The gut microbiota generates numerous small-molecule metabolites during microbial food digestion, playing a vital role in communication between host cells and gut bacteria (Ursell et al., 2014). The metabolites could have beneficial or harmful effects. Over the past decade, more than 300 endogenous metabolites of gut bacteria, including SCFAs, BAs, monoamines, biogenic amines, indole derivatives, phenols, vitamins, branched-chain amino acids (BCAAs), and lipids, have been discovered through non-targeted and targeted metabolomic analyses. Among them, extensive studies have been conducted on the primary endogenous metabolites, including, SCFAs, BAs, BCAAs, and TMAO (monoamines). These metabolites play significant roles in various physiopathologic processes. Furthermore, lipopolysaccharides, produced by Gram-negative bacteria, have also attracted attention for their effect on the host through metabolism-independent signaling pathways(Yang et al., 2021). This highlights the importance of understanding the complex interactions between these metabolites and their potential roles in human health and disease (Li et al., 2018b; Wang et al., 2018).Therefore, this section presents the effect of gut microbial metabolites on the host from the perspective of the immune system.

#### 3.3.1 Short-chain fatty acids

Short-chain fatty acids (SCFAs), or volatile fatty acids, are a class of low-molecular organic fatty acids with approximately two to six carbon atoms. Short-chain fatty acids arise from the fermentation of dietary fiber by microorganisms in the colon (Chen et al., 2020). Short-chain fatty acids mainly include acetic acid, propionic acid, butyric acid, isobutyric acid, valeric acid, isovaleric acid, caproic acid, and isocaproic acid. The major metabolic products include SCFAs acetate (C(2)), propionate (C(3)) and butyrate (C(4)) (Schwiertz et al., 2010; Vinolo et al., 2011). The type and amount of SCFAs depend on the composition and fermentative ability of the gut microbiota, digestion time, host-microbe metabolic flux, and the fiber content of the host food (Parada Venegas et al., 2019; Deleu et al., 2021). Short-chain fatty acids are absorbed by the intestine primarily through monocarboxylate transporters 1 (MCT1) and 4 (MCT4) (Kirat et al., 2006; Sivaprakasam et al., 2017). The SCFAs promote cell growth, improve intestinal function, and influence cardiovascular metabolism. In addition, SCFAs act as mediators of the immune response and promote the production of anti-inflammatory cytokines by peripheral blood monocytes (Asarat et al., 2016). Depletion of gut microbiota by antibiotics decreased immune cell composition and impaired repair after myocardial infarction, while supplementation with SCFAs or *Lactobacillus* probiotics restored these effects. This highlights the importance of gut microbiota-derived SCFAs in modulating pathological outcomes after myocardial infarction and potentially impacting human health and disease as a whole (Tang et al., 2019a).

Short-chain fatty acids regulate the effector functions of CD8^+^ T cells by activating the G protein-coupled receptor 41(GPR41)(Vinolo et al., 2011). In addition, SCFAs can activate GPR43 or the free fatty acid receptor (FFAR 2) in peripheral adipose tissue, thereby regulating insulin sensitivity, promoting glucagon-like peptide 1 (GLP-1) release from stimulated L cells and regulating inflammation (Priyadarshini et al., 2016). One study proposed that SCFAs exert their effects on leukocytes and endothelial cells *via* two known mechanisms, i.e., activating GPR41 and GPR43 and inhibiting the histone deacetylase (HDAC) activity (Vinolo et al., 2011). Butyric acid and propionic acid inhibit HDAC activity, LPS-induced secretion of inflammatory mediators by macrophages, and macrophage reactivity, as well as exert anti-inflammation effects in mice (Li et al., 2018a; Yao et al., 2022). In addition, acetic acid induces immunoglobulin A (IgA) production in the intestines of mice and maintains a relatively stable immune system (Mei et al., 2022). However, excessive production of acetic acid can induce colitis (Sharon and Stenson, 1985; Uraz et al., 2013). Furthermore, butyric acid can attenuate inflammation by reducing macrophage adhesion and migration (Smith et al., 1998), hence reducing the production of IL-6 and IL-12, and increasing IL-10 (Park et al., 2022). Moreover, butyric acid and propionic acid produced by the gut microbiota can promote the differentiation of peripheral Tregs and maintain immune homeostasis (Kim et al., 2014; Asarat et al., 2016).

Taken together, SCFAs modulate the immune system and alleviate inflammation.

#### 3.3.2 Bile acids

Bile acids (BAs) promote the emulsification of fats, thus increasing the surface area for pancreatic lipase and improving the solubility of lipids by forming mixed micelles (Amara et al., 2019). Bile acids facilitate the absorption of lipids in the small intestine. Clinical studies and animal experiments have shown that BAs, particularly secondary BAs (SBAs) generated during bacterial metabolism of BAs, can influence intestinal inflammation (Schirmer et al., 2019; Cai et al., 2022). The related gut microbiota mainly comprises Lactic acid bacteria, *Enterobacteriaceae*, and *Enterococci* bacteria, which can deconjugate BAs from taurine or glycine to produce deoxycholic acid (DCA) and lithocholic acid (LCA).

Bile acids are important signaling molecules, which regulate host metabolism and energy homeostasis, and affect innate immunity (Hang et al., 2019; Han et al., 2022). One recent study revealed that gut microbiota and LCA could regulate the host immune response by directly altering the balance between Th17 and Tregs (Hang et al., 2019). Previous studies have shown that SBAs could exert pro-inflammatory effects at high concentrations (Hang et al., 2019; Han et al., 2022). Digoxin was identified as the first Th17 cytostatic agent that binds to the retinoic acid-related orphan receptor-gamma-t (RORγT) (Karaś et al., 2018). After digoxin identification, other structurally related cholesterol derivatives have been identified as modulators of RORγT. Bile acids are cholesterol metabolites present in the intestine. Bile acids control Th17 by targeting RORγT activity (Song et al., 2020). BAs can inhibit macrophage function by activating BA receptors and promoting the differentiation of Tregs. The BAs receptors include nuclear receptors such as the farnesoid X receptor (FXR), the G protein-coupled bile acid receptor 1 (GPBAR1/TGR5), pregnane X receptor (PXR), sphingosine 1-phosphate receptor 2 (S1PR2), and other membrane receptors (Wang et al., 2011; Hu et al., 2022; Yao et al., 2022). By activating these receptors, BAs inhibit the overgrowth of intestinal bacteria, thus protecting against visceral infection.

Taken together, BAs may have pro-inflammatory or anti-inflammatory effects, depending on the type and concentration of bile acids.

#### 3.3.3 Branched-chain amino acids

Branched-chain amino acids (BCAAs) have an aliphatic side chain and a branch (a central carbon atom bound to three or more additional carbon atoms). Leucine, isoleucine, and valine are three naturally occurring proteinogenic BCAAs (Lynch et al., 2014). These amino acids cannot be synthesized in animals. However, they are synthesized in bacteria, plants, and fungi (Neinast et al., 2019). The metabolism of BCAAs involves several complex enzymatic reactions. Furthermore, branched-chain amino acids affect cellular metabolism.

A previous study revealed that dietary supplementation of BCAAs in middle-aged mice is associated with increased mitochondrial formation and bioenergetics as well as reduced ROS production, which could prevent aging and promote survival (D’Antona et al., 2010). Furthermore, BCAAs may inhibit fibrosis by decreasing apoptosis, caspase-3 activity, and oxidative stress in mice (Takegoshi et al., 2017). In addition, BCAAs enhance the immune response of NK cells (Matsumoto et al., 2009) and liver-associated lymphocytes (Kajiwara et al., 1998; Takegoshi et al., 2017). A previous study investigated the potential role of BCAAs in reducing inflammation and improving immune function in athletes and individuals undergoing physical stress. BCAAs serve as signaling molecules. For example, BCAAs can activate the mammalian/mechanistic target of rapamycin complex 1 (mTORC1) (Mann et al., 2021). Previous studies revealed that supplementation of BCAAs reduced inflammation and oxidative stress in athletes, thereby improving their immune function and reducing infection risk (Matsumoto et al., 2009; Kim et al., 2012). However, high plasma levels of BCAAs are associated with inflammation, insulin resistance, and metabolic syndrome (Yoon and Yoon, 2016).

Taken together, the role of BCAAs on inflammation depends on the BCAAs concentration and the individual’s health status.

#### 3.3.4 Trimethylamine-N-Oxide

Trimethylamine-N-Oxide (TMAO) is an intestinal-derived flora-related metabolite synthesized in the liver. It is derived from trimethylamine (TMA), metabolized by the gut microbiota. Some gut microbiota produces trimethylamine lyase, an enzyme that converts dietary choline, betaine, carnitine, and TMA- structured food into TMA (Cho and Caudill, 2017). For example, some bacteria belonging to the phylum Firmicutes, including certain species within the Clostridia class and the *Enterococcus* genus, as well as the *Desulfovibrio* genus from the phylum Proteobacteria, can produce enzymes like choline TMA-lyase, which is involved in the generation of trimethylamine. Trimethylamine is transported to the liver *via* portal circulation, where it is oxidized by flavin monooxygenases 3 (FMO_3_) to produce TMAO (Liu et al., 2020b).

Trimethylamine-N-Oxide promotes monocyte adhesion, increases macrophage infiltration, and promotes foam cell production. It has been shown that TMAO can inhibit the cellular activity of antioxidant enzymes, including superoxide dismutase (SOD) and catalase (CAT), causing a reduction in the antioxidant activity of cells (He et al., 2021). Similarly, TMAO promotes the production of reactive oxygen species (ROS), thus exacerbating oxidative stress (Querio et al., 2019). Animal studies have shown that TMAO induces vascular inflammation by activating the sirtuin 3 -superoxide dismutase 2- mitochondrial ROS (SIRT3‐SOD2‐mtROS) and stimulating *in vitro* and *in vivo* formation of the NLRP3 inflammasome (Chen et al., 2017). In addition, TMAO can induce vascular inflammation by activating the mitogen-activated protein kinase (MAPK) and nuclear factor kappa-B (NF-κB) signaling pathways (Seldin et al., 2016). TMAO also activates the inflammatory response by inducing the expression of IL-6, cyclooxygenase-2 (COX-2), endothelial selectin, and intercellular cell adhesion molecule-1 (ICAM-1), which enhances macrophage adhesion through protein kinase C (PKC) and NF-κB signaling pathway. Furthermore, high serum levels of TMAO can increase the production of tumor necrosis factor-α (TNF⁃α) through the NF⁃κB signaling pathway. A recent study demonstrated that silencing of FMO_3_ was associated with decreased production of TMAO(Schiattarella et al., 2017). TMAO has also been shown to trigger adipose tissue inflammation (Yang et al., 2021).

Taken together, TMAO has pro-inflammatory effects.

#### 3.3.5 Lipopolysaccharides

Endotoxins/LPS are a complex of lipids and polysaccharides. They are structural components of the outer membrane of Gram-negative bacteria such as *Pectinatus*. In addition, LPS determines the diversity of bacterial antigens. During bacterial pathogenesis, lipopolysaccharides trigger inflammation by activating the Toll-like receptor 4 (TLR4) in immune cells and other cell types, including adipocytes and hepatocytes (Neves et al., 2013; Schoeler et al., 2019). As activators of innate immune responses, LPS have a non-negligible role in human immune responses (Neves et al., 2013).

Interactions between LPS and Toll-like receptor 4 (TLR4) on surfaces of immune cells such as macrophages and dendritic cells induces a cascade of signaling events that produce of pro-inflammatory cytokines, such as TNF-α, IL-1β, NF-κB, and IL-6. These cytokines play a crucial role in the recruitment and activation of other immune cells, including neutrophils and NK cells, to sites of infection (Medzhitov and Medzhitov, 2007). Besides activating the innate immune system, LPS influences adaptive immune responses. They improve the antigen-presenting abilities of dendritic cells, which are crucial for initiating and shaping adaptive immune responses. The LPS can also promote T cells activation (CD4^+^ helper and CD8^+^ cytotoxic T cells) against the pathogen (Hoebe et al., 2004). In addition, LPS is useful in modeling inflammation-related diseases, including sepsis and myocarditis by activating the NF-кB signaling pathway (Wang et al., 2019b).

Gut microbiota-produced metabolites, including SCFAs, BAs, BCAAs, TMAO, and LPS can affect the immune system and contribute to development of inflammatory diseases. Additional studies should focus on elucidating the mechanisms underlying these associations and exploring the potential therapeutic interventions targeting these metabolites.

## 4 Myocarditis and the immune system

The role of inflammation in the progression of CVDs has attracted considerable attention. In pathogenesis, IL-1β, IL-6, TNF-α, and interferon-gamma (IFN-γ) are associated with heart inflammation while IL-10, TGFβ, and others are linked to the resolution of inflammation and heart tissue repair. IL-10 mitigates inflammation in the cardiovascular system and exerts protective effects by interacting with SMAD2, p53, HuR, miR-375, and miR-21 pathways (Goswami et al., 2021). Myocarditis is an inflammatory disease that affects myocardium health, and the extent of damage depends on the nature of the pathogen and associated inflammatory responses. Myocarditis is characterized by immune responses specific to the heart and is categorized based on the clinical and histopathological features (**Figure 1**) (Heymans et al., 2016). Experimental mice models have shown the significance of immune cells in myocarditis development (Swirski et al., 2018). The proinflammatory cytokine, IL-1, is crucial in the development of myocardial inflammation (Cavalli et al., 2016). IL-α activates the ‘inflammasome,’ leading to the infiltration of inflammatory cells, processing and release of active IL-1β (Toldo et al., 2014). IL-1β, a highly studied member of the IL-1 cytokine family, is primarily influenced by the functioning of the NLRP3 inflammasome in inflammation (Abbate et al., 2020). This section focuses on two types of myocarditis: Viral myocarditis and autoimmune myocarditis.

### 4.1 Viral myocarditis

Viral myocarditis is a significant cause of heart failure and dilated cardiomyopathy. Viral infection of the myocardium can lead to myocardial cell necrosis. The pathological and physiological mechanisms of viral myocarditis have been investigated using murine models of enterovirus infection, especially coxsackievirus B3 (CVB3) (Lin et al., 2021). The CVB3 cardiomyophilic strain virus (CVB3m) and the CVB3 non-cardiomyophilic strain virus (CVB3o) are variants of the CVB3 standard strain, which can be adapted for different research objectives to achieve the required pathology of myocarditis in specific tissue types (Błyszczuk, 2019).

Viral entry into the myocardium results in three kinds of responses. The acute phase is characterized by viral entry and replication, the subacute phase is characterized by inflammatory cell infiltration, and the chronic phase is characterized by cardiac remodeling. Myocardial injury includes direct injury mediated by viral infections and indirect injury due to secondary immune responses (Henke et al., 1995). The molecular mechanisms underlying injury were described in detail in a review published in 2016. Targeting these virus-encoded proteases may inhibit viral replication and viral direct damage to the myocardium (Fung et al., 2016). The adaptive immune responses begin after the acute and subacute phases of myocarditis (Lin et al., 2021). Opavsky et al. established gene knockout mice CD4^(-/-)^, CD8^(-/-)^, both co-receptors (CD4^(-/-)^ CD8^(-/-)^), or T cells receptor beta chain (TCR beta ^(-/-)^) to investigate the impact of T cell subsets on host susceptibility to CVB3 myocarditis. They found that myocarditis severity in the CD4 knockout group, CD4 and CD8 knockout group, and TCR beta knockout group was lighter than that in the CD8 knockout group. Moreover, IFN-γ levels were elevated while TNF-αlevels were suppressed in CD4 and CD8 knockout mice models (Opavsky et al., 1999). Chemokines are a class of small cytokines or signaling proteins secreted by cells. They can induce nearby responsive cells to directionally migrate towards the source of chemokines. A transgenic study involving mice models revealed that CXC chemokine ligand 10 (CXCL10) was upregulated in the early stages of myocardium infection and inhibited viral replication in the early CVB3 infection stages by recruiting NK cells and promoting IFN-γ expressions. However, in the late infection stages, it did not stimulate the antiviral effects to improve the survival rates of mice (Yuan et al., 2009). Male mice with CVB3-induced myocarditis had myocardial infiltrating macrophages expressing increased markers, including inducible nitric oxide synthase, IL-12, TNF-α, and CD16/32, which are associated with classically activated macrophages (M1) (Li et al., 2009).

### 4.2 Autoimmune myocarditis

Mice models are important in studies on autoimmune diseases (Lincez et al., 2011). Experimental autoimmune myocarditis mice models distinguish between autoimmune phases of viral myocarditis from the acute infection phase of CVB in genetically modified mice (Blyszczuk et al., 2008). Disease severity is classified based on the infiltration extent of inflammatory cells during the peak of inflammation. This model can also be used for other types of myocarditis (Błyszczuk, 2019).

Neu et al. reported that autoimmune myocarditis is often indirectly associated with a viral infection. One possible contributing factor is the release or exposure of cardiac myosin after viral-mediated myocyte damage, which induces autoimmune responses and myocardial inflammation (Neu et al., 1987). Pdcd1^-/-^Ctla4^+/-^ mice spontaneously develop fulminant myocarditis. Therefore, Axelrod et al. performed single-cell sequencing and single-cell TCR sequencing of immune cells infiltrating these myocarditis tissues and found that CD8^+^ T cells were significantly increased in number and showed clonal expansions. Myocarditis did not develop when CD8^+^ T cells were removed from these mice. When CD4^+^ T cells were the only ones to be removed, myocarditis incidences did not change. When CD8^+^ T cells from Pdcd1^-/-^Ctla4^+/-^ mice were adoptively transferred to Rag1^-/-^ mice, the recipient mice developed myocarditis after two months. Therefore, CD8^+^ T cells are highly involved in fulminant myocarditis development (Axelrod et al., 2022). A recent study also found that macrophage migration is a significant histopathological feature of myocarditis, indicating that macrophages are potential therapeutic targets for this disease (Toita et al., 2021).

## 5 Gut microbiota and myocarditis, inflammatory cardiomyopathy

In previous discussions, we highlighted the strong associations between gut microbiota and the immune system. Furthermore, we elucidated the role of the immune system in myocarditis development. In this section, we assess the relationship between gut microbiota and myocarditis.

It has been demonstrated that FMT, a method used to restore gut microbial homeostasis, can improve myocardial damage in myocarditis mice (Hu et al., 2019). Zhang et al. investigated the significance of GLP-1 receptor agonists in alleviating autoimmune myocarditis by modulating gut microbiota (Zhang Wenyong, 2019). In spontaneous autoimmune myocarditis mice, gut microbiota promotes disease and interacts with MYH6-specific CD4^+^ T cells. Cruz et al. found that commensal Bacteroides produce an MYH6 mimic, β-galactosidase, which can lead to the activation of cross-reactive Th17 cells, ultimately causing inflammatory cardiomyopathy in individuals with a genetic susceptibility to this condition. Antibiotics reduce inflammation and prevent death in these mice. Acute myocarditis patients have increased anti-Bacteroides IgG and cross-reactive T cell activation (Gil-Cruz et al., 2019). These findings provide a better understanding of the mechanisms underlying the interaction between the microbiome and the immune system in autoimmune diseases. In the meanwhile, these findings also offer fresh insights into potential strategies for preventing and treating inflammatory cardiomyopathy. Additionally, the European Heart Journal reported Cruz et al’s study. The study focused on the largely unknown processes that cause myocarditis, revealing that gut bacteria composition promotes the development of myocarditis and inflammatory cardiomyopathy. These findings provide valuable insights into the role of gut microbiota in myocarditis, paving the way for future research and potential treatments targeting the gut-heart axis (Ozkan, 2022). Immune checkpoint inhibitors (ICIs) have revolutionized cancer treatment but can also initiate autoimmune diseases, including cardiomyopathy. Although antibiotics may counteract cardiomyopathy by eliminating cross-reacting bacteria, they could also impede ICI efficacy, as gut bacteria play a crucial role in ICI efficacy. A more targeted approach, including phage therapy, could be considered to specifically eradicate immune-mimicking gut commensals without compromising immunotherapy outcomes (Mandelbaum et al., 2020). Han et al. performed HeLa cellular experiments and revealed that gut microbiota metabolites-BAs can inhibit viral replication and attenuate endoplasmic reticulum stress-induced cell death (Han et al., 2018). Barin et al. revealed that enteric microorganisms play a role in determining the susceptibility of mice to the model of experimental autoimmune myocarditis (EAM) and its sequela, inflammatory dilated cardiomyopathy (Barin et al., 2017). Myopericarditis is an inflammatory heart condition involving the pericardium and myocardium, which has been linked to gut microbiota and its metabolites. Piccioni et al. explored the role of gut microbiota in myopericarditis, particularly in relation to the cardiovascular implications of COVID-19, suggesting that microbiota modulation may be a novel approach for preventing or treating inflammatory cardiomyopathies (Piccioni et al., 2021). One study investigated the causal relationship between gut microbiota, their metabolites, and heart failure and its risk factors using Mendelian randomization analysis. Genetic predictions revealed that with every 1-unit increase in *Shigella* concentration, the relative risk for myocarditis increases by 38.1%. These findings may guide future microbiome-based interventions in clinical trials (Luo et al., 2022).

In summary, gut microbiota dysbiosis may be implicated in myocarditis pathogenesis. Targeting gut microbiota provides novel clinically relevant strategies for myocarditis treatment. The mechanisms by which gut microbiota promote myocarditis are complex and involve dysregulation of the immune system, inflammation, endothelial dysfunction, and metabolism. By revealing the role of gut microbiota in the development and progression of myocarditis, we can gain insight into the disease’s pathophysiology and devise new therapeutic strategies.

## 6 Current strategies for modulating gut microbiota

**Figure 4:**
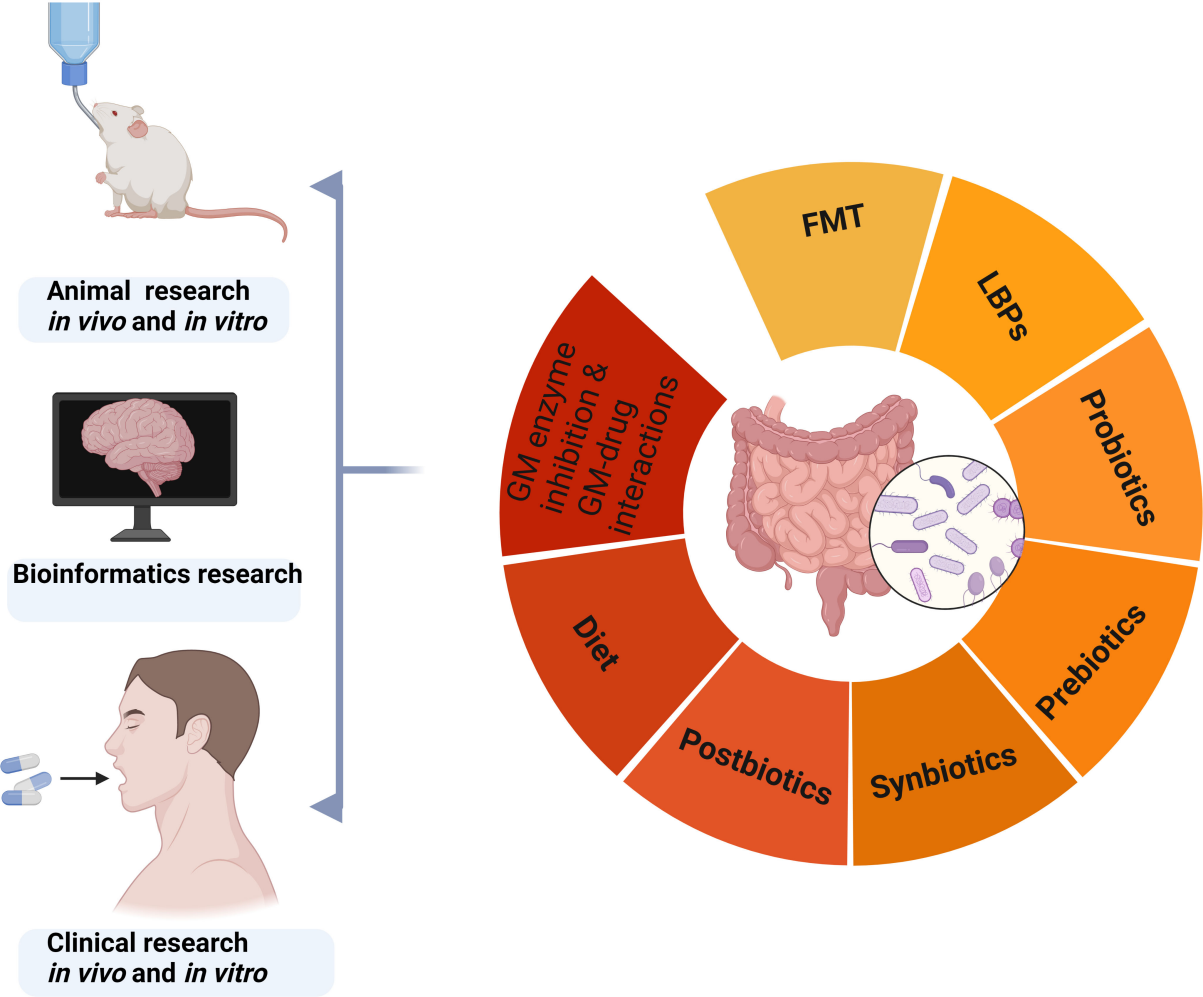
Strategies for regulating gut microbiota based on animal, bioinformatics, and clinical research. GM, gut microbiota; FMT, fecal microbiota transplantation; LBPs, live biotherapeutic productions.

The current strategies for modulating gut microbiota include fecal microbiota transplantation (FMT), live biotherapeutic productions (LBPs), probiotics, prebiotics, symbiotics, dietary interventions, gut microbiota enzyme inhibition, and microbial-drug interactions (**Figure 4**) (Schneiderhan et al., 2016; Adak and Khan, 2019; Wargo, 2020).

### 6.1 Fecal microbiota transplantation

FMT, a natural microbial ecosystem in feces, involves the transfer of fecal material from a healthy donor to a recipient to restore a healthy gut microbiota composition and functions (Wang et al., 2019a). Infant colonization by a specific microbial community, largely originating from the mother, is a natural process that rapidly occurs after birth and is influenced by the delivery mode (Fuentes and de Vos, 2016). As early as the 4^th^ century BC, Chinese medical books recorded the use of fecal preparations to treat gastrointestinal diseases (Wargo, 2020). FMT can effectively treat recurrent *Clostridioides difficile* infections, and there is a growing interest in the use of FMT in other diseases, including inflammatory bowel disease and metabolic syndromes (Davidovics et al., 2019). The success rate of FMT relies on the composition of the recipient’s microbiome and the interplay between the microbiomes of the donor and recipient at both taxonomic and functional levels (Kazemian et al., 2020). FMT can provide a diverse range of microbial communities which play an important role in restoring functional redundancy of gut microbiota. However, there exist limitations in terms of reproducibility, whereas factors including safety, donor/recipient considerations, dosing, and administration route should be taken into account when considering FMT. Studies on the use of FMT, specifically in the context of myocarditis, are limited (Hu et al., 2019). Further, the safety and efficacy of FMT in myocarditis treatment should be investigated.

### 6.2 Live biotherapeutic productions, probiotics, and synbiotics

1) The LBPs are a type of therapeutic agent comprising many live microorganisms, typically bacteria, ingested or applied to the body for disease prevention and treatment (Charbonneau et al., 2020). They are also referred to as “live biotherapeutics” or “live biotherapeutic agents” (Dhansekaran and Sankaranarayanan, 2021). They differ from probiotics or prebiotics in that they are specifically designed to deliver a therapeutic effect (Dreher-Lesnick et al., 2017; Charbonneau et al., 2020). They are produced under strict manufacturing processes to ensure the viability and efficacy of bacteria when administered to the patient. Some clinical trials are assessing the efficacy of this strategy of regulating gut microbiota in cancer immunotherapy (Wargo, 2020). The potential of LBPs to treat various conditions, including gastrointestinal, metabolic, and immune system disorders is being investigated. A previous review outlined the factors to be considered during the design and development of genetically engineered LBPs to ensure compliance with regulatory standards and gain acceptance from patients (Charbonneau et al., 2020).

2) Antibiotics are drugs that can kill or inhibit the growth of bacteria. Excess or improper use of antibiotics can lead to gut microbiota dysbiosis (Gough, 2022). Probiotic supplementation is a treatment approach that can promote gut microbiota recovery. Probiotics are live microorganisms such as *Bifidobacterium, Saccharomyces*, Lactic acid bacteria*, Lactobacillus acidophilus, Actinomycetes*, and *Lactobacillus rhamnosus* with health benefits upon consumption (Hill et al., 2014). Probiotic supplementation reduces myocardial hypertrophy and heart failure following myocardial infarction in rat models (Gan et al., 2014). Intestinal stem cell regeneration was accelerated by *Lactobacillus rhamnosus GG*, which promoted colonic barrier recovery in septic mice (Chen et al., 2023). *In vitro*, anti-inflammatory activities of probiotic supernatants are unique, since they can modulate interleukin 1β (IL-1β), IL-6, TNF-α, and IL-10 production in human macrophages in distinct approaches (De Marco et al., 2018). Probiotic supplementation is considered safe for individuals with functional immune systems (Vallianou et al., 2020). Still, they may induce immune responses, require cold storage, and cannot be used with antibiotics (Suez et al., 2019).

3) Symbiotics are combinations of probiotics and prebiotics with synergistic effects on gut microbiota composition and function. They are developed to address the potential challenges associated with probiotics survival in the gastrointestinal tract (Rioux et al., 2005). Consumption of yogurt and fruits may have combined health benefits due to their potential prebiotic and probiotic effects (Fernandez and Marette, 2017).

### 6.3 Prebiotics, postbiotics, and dietary interventions

1) Prebiotics are nondigestible food ingredients that selectively stimulate the growth and activities of beneficial gut microbiota. They primarily consist of bifidogenic, non-digestible oligosaccharides, such as inulin, its hydrolysis product oligofructose, and (trans) galactooligosaccharides (de Vrese and Schrezenmeir, 2008). Prebiotics include nutritional supplements that promote gut microbiota proliferation. The natural sources of prebiotics include beans, cereals, and soybean among others (Kerry et al., 2018). They may reduce inflammatory responses and improve cardiac functions by regulating gut microbiota. Similar to findings from studies involving adult studies, prebiotic effects in infant nutrition results in significant alterations in gut microbiota composition, with a notable increase in *Bifidobacteria* levels in fecal matter (Roberfroid et al., 2010).

2) Postbiotics, gaining attention as health-promoting agents, are beneficial compounds produced through the metabolic activities of microorganisms, particularly probiotics. These diverse substances, including cell wall components, SCFAs, and enzymes, have various effects on the host, such as modulating the immune system, improving gut barrier function, and inhibiting pathogenic bacteria growth (Żółkiewicz et al., 2020); Tsilingiri and Rescigno, 2013). As a stable and safe alternative or complement to traditional probiotics, postbiotics show promise in maintaining and improving human health, necessitating additional research to optimize their production and develop effective therapies.

3) Food plays a vital role in shaping gut microbiota composition and diversity. Dietary interventions, including the Mediterranean diet and Dietary Approaches to Stop Hypertension diet, have beneficial effects on gut microbiota composition and cardiovascular health (Merra et al., 2020; Drapkina et al., 2022). The Mediterranean diet, rich in fruits, vegetables, whole grains, legumes, fish, and olive oil, improves gut microbiota composition, reduces frailty and improves health status (Ghosh et al., 2020). The Dietary Approaches to Stop Hypertension (DASH) diet, rich in fruits, vegetables, whole grains, and low-fat dairy products, reduces blood pressure and improves cardiovascular health (Maifeld et al., 2021). A Western-style diet, rich in saturated fats, salt, and sugar, reduces gut microbiota diversity which increases the prevalence of inflammatory disease (Statovci et al., 2017). Plant-based diets, rich in fibers, are associated with increased gut microbiota diversity and reduced cardiovascular disease risks (Satija and Hu, 2018; Losno et al., 2021).

### 6.4 Microbial-drugs interactions and gut microbial enzyme inhibition

1) The gut microbiome plays a crucial role in mediating host-environment interactions and exhibits a complex bidirectional relationship with non-antibiotic drugs. This intricate interplay involves the microbiome influencing drug efficacy and toxicity, while simultaneously being affected by the drugs themselves, a phenomenon known as pharmacomicrobiomics (Weersma et al., 2020). We review the relevant research on the interactions between gut microbiota and cardiovascular medications. Silva et al. discovered that statin therapy is a key covariate affecting gut microbiome diversity by analyzing the data from 888 volunteers in the Body Mass Index Spectrum cohort from the MetaCardis project. In patients with ACS under statin medications, the potentially pathogenic bacteria in the gut are reduced, with a better prognosis. Analysis of fecal samples suggests that the gut microbial communities of obese individuals taking cholesterol-lowering statin medications are “healthier” than expected, suggesting that the potential beneficial effects of statins on gut microbiota open up new prospects for disease treatment (Vieira-Silva et al., 2020). Yang et al. discovered a previously unrecognized mechanism in which the human commensal bacteria, *Coprococcus* comes, catabolizes ester ACE inhibitors in the gut, reducing their antihypertensive effects. The findings revealed that gut microbiota may play a role in the efficacy of antihypertensive medications, which could help explain why some individuals remain resistant to treatment (Yang et al., 2022a). Another study found that liraglutide could treat patients with type 2 diabetes mellitus by targeting the gut microbiota (Shang et al., 2021)

In clinical practice, traditional Chinese medicine (TCM) is orally administered and bidirectionally interacts with the gut microbiota. These interactions have two primary effects: (1) Enhancing effects: TCM modulates gut microbiota composition and metabolism, improving host health while the microbiota enhances TCM bioavailability. (2) Inhibitory effects: Some TCM constituents can weaken gut microbiota metabolic functions, and certain microbes may inhibit TCM absorption and metabolism (Zhu et al., 2023). A recent review revealed that numerous natural molecules (e.g., apigenin, berberine, and quercetin) and plant extracts can effectively alleviate experimental autoimmune myocarditis. Key anti-myocarditis mechanisms include the upregulation of Th1-type cytokines, the elevation of Th2-type cytokines (IL-4 and IL-10), mitigation of oxidative stress, modulation of mitogen-activated protein kinase signaling pathways, and increased sarco-endoplasmic reticulum Ca2^+^-ATPase levels (Javadi and Sahebkar, 2017). These molecules and extracts can alter the composition and abundance of gut microbiota, suggesting that they hold great potential as treatments that target gut microbiota.

The use of antibiotics can not only fight against pathogenic bacteria, but also affect the intestinal symbiotic flora. Compared to other antibiotics, the gut symbiotic bacteria are more sensitive to macrolides and tetracyclines. Some detoxifying agents protect the gut symbiotic bacteria from antibiotic damage (Maier et al., 2021). Haak et al. found that a one-week course of combined broad-spectrum antibiotics (ciprofloxacin, vancomycin, and metronidazole) has a profound and long-lasting impact on the gut microbiota of healthy humans, causing loss of diversity and shifts in community composition. Although the microbiota showed a remarkable return towards baseline after 8-31 months, the community composition often remained altered, with the long-term consequences remaining largely unknown (Haak et al., 2019).

2) Moreover, Mamic et al. summed up the application of gut microbiome in heart failure and its comorbidities. They highlighted that targeting gut microbial enzymes, which are not found in the host, is a promising approach to overcoming these challenges. The study focuses on the TMAO meta-organismal pathway and suggests that 3,3-dimethy-1-lbutanol, a natural inhibitor of TMA lyases, may be a non-lethal and elegant strategy to target this pathway. In mice fed a high choline diet, administration of 3,3-dimethy-1-lbutanol resulted in decreased circulating TMAO levels, decreased foam cell formation, and fewer atherosclerotic plaques. Targeted inhibition of microbial choline-TMAO conversion was also evaluated in a pressure overload mice model of heart failure, where it improved cardiac remodeling and cardiac function. The study proposes that both pharmacologic modification of the TMAO biosynthetic pathway and targeted dietary interventions may be viable strategies for modulating the pathogenesis and progression of heart failure. However, further human studies are necessary to evaluate the feasibility and efficacy of this approach (Mamic et al., 2021).

Gut microbiota dysbiosis is implicated in the pathogenesis of many diseases. The LBPs, FMT, pre/probiotics, postbiotics, synbiotics, and dietary interventions have the potential for disease prevention or treatment by modulating gut microbiota. The future of promoting overall health and treating diseases by regulating intestinal microorganisms is becoming clearer, and more strategies and methods to regulate gut microbiota are known (Wargo, 2020). The various approaches have shown promising results in animal studies, however, their effectiveness and safety for myocarditis treatment should be investigated further. Elucidating the mechanisms by which gut microbiota contribute to myocarditis pathogenesis may lead to the development of novel therapeutic approaches targeting the gut microbiota.

## 7 Challenges and future direction of targeted gut microbiota in myocarditis treatment

Myocarditis is a serious inflammatory disease of the heart muscles that can lead to heart failure and sudden cardiac death. The current treatment options for myocarditis are not fully effective, and there is a growing interest to understand the efficacy of targeted gut microbiome therapy (Hu et al., 2019). However, this approach has several challenges, including low efficacy of current treatment methods and the need for personalized treatment (Schneiderhan et al., 2016). Therefore, future research is needed to identify new therapeutic targets. Targeted gut microbiome therapy for myocarditis aims to restore the balance of bacteria in the gut and reducing the production of pro-inflammatory cytokines. However, the complex and diverse nature of the gut microbiome presents challenges in developing targeted therapies, therefore, studies should investigate its roles in disease development to identify effective treatment strategies.

One possible approach for targeted gut microbiome therapy is the use of gut microbiota-related strategies, including probiotics and FMT. Probiotics can reduce inflammation and improve immune functions, which may be beneficial in myocarditis treatment. FMT can help in restoring the balance of bacteria in the gut to reduce inflammation (Hu et al., 2019). Elucidating the mechanisms of enterobacteria in myocarditis will inform the development of enterobacteria-targeting drugs. However, a limited number of studies have investigated the relationship between enterobacteria and myocarditis. Herein, we summarize major studies with regard to myocarditis treatment, which may inspire further research design and direction (**Table 1**).

**Table 1:** Exploration of the treatment of myocarditis in previous studies.

Interventions	Classification	Research design	Targets	Results	References
**Nanoparticle-encapsulated siRNA**	Acute autoimmune myocarditis	Human studies; *In vivo*, and *in vitro*, A/J mice model	CCR_2_	↓ Ly6C^high^ monocytes	(Leuschner et al., 2015)
**PSL-G**	Experimental autoimmune myocarditis	*In vivo* and *in vitro, A/J* mice model	Macrophages	↓ Pro-inflammatory cytokines (e.g., IL-1α, IL-6, and TNF-α); ↑ Anti-inflammatory cytokine IL-10; ↑ Macrophage polarization: from the pro-inflammatory M1 phenotype to the anti-inflammatory M2 phenotype	(Toita et al., 2021)
**Silencing of microRNA-30a-5p**	Viral myocarditis	*In vivo* and *in vitro*, BALB/c mice model	SOCS1	↓ M1 polarization of macrophages;	(Zhang et al., 2021a)
**Anakinra**	Fulminant myocarditis	*In vivo*, patient	IL-1 receptor	↓ Circulating neutrophils	(Cavalli et al., 2016)
**Fructus Amomi Cardamomi Extract**	CVB3 myocarditis	*In vivo* and *in vitro*, mice model	Undisclosed	↓ *Enterovirus* replication; ↓Myocarditis damage	(Lee et al., 2016)
**Lithium chloride**	CVB3 myocarditis	*In vivo* and *in vitro*, mice model	Undisclosed	↓Virus-triggered inflammatory responses; ↓CVB3 replication	(Zhao et al., 2020)
**Zinc finger antiviral protein**	CVB3 myocarditis	*In vivo* and *in vitro*, BALB/c mice model	Viral RNA	↓ Viral replication ↓Cardiac inflammatory cytokine production	(Li et al., 2015)
**Tripartite motif-containing 21**	CVB3 myocarditis	*In vivo* and *in vitro*,BALB/c mice model	Mitochondrial antiviral signaling protein	↓CVB3 replication ↑IFN-β	(Zhang et al., 2012; Liu et al., 2018; Xue et al., 2018)
**FMT**	Experimental autoimmune myocarditis	*In vivo*, male BALB/c mice	Gut microbiota	Rebalancing the microbiota composition; ↓Inflammatory infiltration	(Hu et al., 2019)
**Liraglutide**	Experimental autoimmune myocarditis	*In vivo*, male BALB/c mice	Gut microbiota and immuse system	↓TNF-α、IL-1β、MCP-1	(Zhang Wenyong, 2019)
**Leonurine**	LPS-induced myocarditis	*In vivo* and *in vitro*, C57BL/6 mice	NF-кB signaling pathway	↑Cardiac function ↓Cardiomyocyte apoptosis	(Wang et al., 2019b)
**Myricetin**	Experimental autoimmune myocarditis	*In vivo and in vitro*, male BALB/c *mice*	The autoimmune response specific to myocardium and the expression of MCP-1	↓Serum anti-cardiac myosin antibody, IgG, IgM levels, and the Th17 cells. ↓MCP-1, phospho (p)-p65, p-c-Jun and Act1/TRAF6/TAK1 ↑Tregs	(Nie et al., 2023)

CCR_2_, chemokine (C-C motif) receptor 2; PSL-G, phosphatidylserine liposomes conjugated with protein G; CVB3, coxsackievirus B3; FMT, fecal microbiota transplantation; IFN, interferon; Ig, immunoglobulin; IL, interleukin; M1, M1 phenotype macrophages; MCP-1, monocyte chemoattractant protein-1; Th17 cells, T helper 17 cells; TNF, tumor necrosis factor; Tregs, regulatory T cells; SOCS1, suppressor of cytokine signaling 1; si-RNA, small interfering RNA.

### 7.1 Limitations of treatment methods

The current treatment options for myocarditis are limited, and there is a need for new approaches to improve outcomes. Targeted gut microbiome therapy is a promising approach; however, it is still in the early developmental stages, and more research is necessary to determine its efficacy and safety. Several challenges and limitations to the development of gut microbiota-targeted therapies are defined by alternatives, such as FMT, and LBPs, as well as by the lack of a clear mechanistic understanding of disease pathophysiology. Another challenge is the lack of knowledge about the roles of specific bacteria in myocarditis pathogenesis (Mandelbaum et al., 2020). Although studies have reported that certain bacteria may be involved in the development of this condition, it is still unclear which bacteria are most important and how they interact with the host immune system (Hu et al., 2019). This limits the capacity to develop targeted therapies that can effectively treat myocarditis.

### 7.2 Personalized treatment and precision medicine

One of the challenges of targeted gut microbiome therapy is the need for personalized treatment. The gut microbiome is highly individualized, and bacterial composition can vary widely from person to person, therefore, a one-size-fits-all approach to treatment is unlikely to be effective. Instead, personalized treatment plans that take into account the specific bacteria present in each patient’s gut microbiome are needed (Wargo, 2020). Precision medicine approaches, such as genomics, metabolomics, and various omics techniques can help in identifying specific bacterial strains that are associated with myocarditis and develop personalized treatment plans based on these findings (Caesar et al., 2021).

### 7.3 Future directions and prospects

Despite the challenges of targeted gut microbiome therapy, it holds great promise in myocarditis treatment. One direction for future research is to identify new targets for therapy based on better understanding of interactions between the gut microbiome and the host immune system. Studies should aim at investigating the etiology, pathogenesis, and gender differences of myocarditis (Tschöpe et al., 2021).

Moreover, it’s possible to apply machine learning and artificial intelligence to analyze gut microbiome data, which can enhance our understanding of the connection between the gut microbiome and myocarditis. By adopting this innovative approach, we can identify previously unknown therapeutic targets and create more personalized treatment plans for individual patients (Loganathan and Priya Doss, 2022).

There is a need for personalized treatment plans based on specific bacteria present in each patient’s gut microbiome (Behrouzi et al., 2019). Targeted gut microbiome therapy has the potential to revolutionize myocarditis treatment and improve disease outcomes. There is a need for large-scale animal and clinical trials to evaluate the safety and efficacy of targeted gut microbiome therapy in myocarditis treatment. Moreover, studies should also explore the optimal timing and duration of treatment and assess the long-term effects of this therapy on patient outcomes.

## 8 Conclusion

Myocarditis and inflammatory cardiomyopathy present considerable threats to human life and well-being by causing inflammation and damage to the heart muscle, potentially resulting in severe complications including heart failure, arrhythmias, and even sudden cardiac death. Nonetheless, treatment options for myocarditis remain limited and research efforts face substantial challenges. The gut microbiota is a critical player in the regulation of immune responses and the maintenance of cardiovascular health. Gut microbiota dysbiosis is implicated in myocarditis development and progression, therefore, gut microbiota modulation may have potential therapeutic effects for this disease. Targeting the gut microbiota through interventions such as drugs, probiotics, prebiotics, symbiotics, antibiotics, FMT, and diet represent promising strategies for myocarditis treatment. Additional investigations are essential to understand the underlying mechanisms through which imbalances in gut microbiota promote myocarditis development. The findings will enable the identification of optimal strategies for targeting gut microbiota in the treatment and management of myocarditis.

## Author contributions

JW and XZ wrote the first draft of the manuscript. XY, HY, and MB revised the manuscript. ZWZ, ZZ, JF, JH, JL, HZ, ZZ, WY, and XW made the figures and table. YW and QT critically revised the manuscript. All authors contributed to the article and approved the submitted version.

## Glossary

**Table:**

ACS	acute coronary syndrome
AF	atrial fibrillation
ApoE	apolipoprotein E
BAs	bile acids
BCAAs	branched-chain amino acids
CAT	catalase
CVDs	cardiovascular diseases
COVID-19	coronavirus disease 2019
CHD	coronary heart disease
COX-2	cyclooxygenase-2
DCA	deoxycholic acid
DASH	Dietary Approaches to Stop Hypertension
FMT	fecal microbiota transplantation
FMO3	monooxygenases 3
FXR	farnesoid X receptor
GPR	G protein-coupled receptor
GM	gut microbiota
HF	heart failure
HDAC	histone deacetylase
LCA	lithocholic acid
Ig	immunoglobulin
IL	interleukin
IFN	interferon
LPS	lipopolysaccharide
LBPs	live biotherapeutic productions
mTORC1	mammalian/mechanistic target of rapamycin complex 1
MCT	monocarboxylate transporters
MYH6	myosin heavy chain 6
NK	natural killer
NLRP3	NOD-like receptor family pyrin domain containing 3
PXR	pregnane X receptor
PKC	protein kinase C
RORγT	retinoic acid-related orphan receptor-gamma-t
ROS	reactive oxygen species
SCFAs	short-chain fatty acids
S1PR2	sphingosine 1-phosphate receptor 2
SOD	superoxide dismutase
TMAO	trimethylamine-N-oxide
Tregs	regulatory T cells
TNF	tumor necrosis factor
TLR4	toll-like receptor 4
TCM	traditional Chinese medicine
